# Supplementary material for: Ultrafast valence to non-valence excited state dynamics in a common anionic chromophore
Source: Nat Commun. 2019 Dec 20;10:5820. doi: 10.1038/s41467-019-13819-6 (PMC6925192; doi:10.1038/s41467-019-13819-6)
Supplement: Supplementary file 1 — Supplementary Information [file 41467_2019_13819_MOESM1_ESM.pdf]

Supplementary Information

Ultrafast valence to non-valence excited state dynamics in a common anionic chromophore

Bull et al.

Supplementary Note 1

Vibrational frequencies (in cm^{-1}) for the S_0 and $S_1(\pi\pi^*)$ electronic states calculated at the ωB97X-D/aug-cc-pVDZ level of theory. Mode ν_3 = 88 cm^{-1} is the principal Franck-Condon active mode upon photoexcitation and the displacement vectors for this mode are shown in Figure 4**a** in the paper. Note, the $S_1(\pi\pi^*)$ frequencies corresponds to the local minimum energy near the Franck-Condon region, not the twisted intermediate.

Mode, ν	S_0	$S_1(\pi\pi^*)$
1	49	43
2	69	62
3	77	88
4	106	108
5	151	156
6	189	190
7	208	206
8	234	239
9	270	263
10	355	345
11	370	351
12	445	390
13	454	462
14	511	514
15	517	528
16	530	532
17	640	636
18	719	731
19	740	754
20	779	800
21	801	802
22	814	814
23	843	843
24	850	898
25	872	944
26	964	973
27	976	990
28	990	1011
29	994	1031
30	1026	1048
31	1097	1080
32	1113	1115
33	1165	1162
34	1171	1172
35	1184	1204

36	1205	1219
37	1233	1242
38	1269	1280
39	1316	1308
40	1341	1337
41	1370	1365
42	1446	1448
43	1461	1457
44	1466	1466
45	1480	1474
46	1516	1475
47	1549	1541
48	1598	1584
49	1629	1653
50	1689	1681
51	1752	1791
52	3041	3055
53	3117	3142
54	3147	3180
55	3152	3187
56	3152	3194
57	3165	3201
58	3189	3224
59	3193	3228
60	3222	3247
